# Assessing the role of Rv1222 (RseA) as an anti-sigma factor of the *Mycobacterium tuberculosis* extracytoplasmic sigma factor SigE

**DOI:** 10.1038/s41598-019-41183-4

**Published:** 2019-03-14

**Authors:** Francesca Boldrin, Laura Cioetto Mazzabò, Saber Anoosheh, Giorgio Palù, Luc Gaudreau, Riccardo Manganelli, Roberta Provvedi

**Affiliations:** 1 Department of Molecular Medicine, University of Padova, Padova, Italy; 2 Département de biologie, Université de Sherbrooke, Sherbrooke, QC J1K 2R1 Canada; 3 Department of Biology, University of Padova, Padova, Italy; 4 Present Address: Institute of Infectious Disease and Molecular Medicine UCT, Cape Town, South Africa

**Keywords:** Transcriptional regulatory elements, Bacterial genes

## Abstract

σ^E^ is one of the 13 sigma factors encoded by the *Mycobacterium tuberculosis* chromosome, and its involvement in stress response and virulence has been extensively characterized. Several sigma factors are post-translationally regulated by proteins named anti-sigma factors, which prevent their binding to RNA polymerase. Rv1222 (RseA), whose gene lays immediately downstream *sigE*, has been proposed in the past as the σ^E^-specific anti sigma factor. However, its role as anti-sigma factor was recently challenged and a new mechanism of action was hypothesized predicting RseA binding to RNA polymerase and DNA to slow down RNA transcription in a not specific way. In this manuscript, using specific *M. tuberculosis* mutants, we showed that by changing the levels of RseA expression, *M. tuberculosis* growth rate does not change (as hypothesized in case of non-specific decrease of RNA transcription) and has an impact only on the transcription level of genes whose transcriptional control is under σ^E^, supporting a direct role of RseA as a specific anti-σ^E^ factor.

## Introduction

During the infectious process, *M. tuberculosis* faces many different stressful environments; therefore, a rapid adaptation to changing conditions is key for this microorganism. Among the different strategies that can be employed by the bacterium to survive and adapt itself to a hostile environment, specific transcriptional regulation mediated by sigma factors plays a relevant role. Sigma (σ) factors are small interchangeable subunits of the RNA polymerase (RNAP) holoenzyme required to initiate transcription and determine the promoter specificity of the enzyme through the recognition of specific −35 and −10 consensus sequences. They can undergo different regulatory mechanisms at transcriptional, post-transcriptional, translational and post-translational levels^1^. Post-translational regulation is usually due to anti-σ factors, which are proteins that specifically bind to σ factors preventing their interaction with the RNA core enzyme. Specific environmental signals can then cause disruption of such an interaction, leading to the release of the σ factor^2^. The genome of *M. tuberculosis* encodes 13 σ factors^3^ that represent the highest number of σ factors per Mb among obligate pathogens^4^.

σ^E^ is an alternative σ factor of *M. tuberculosis* belonging to the extra cellular function (ECF) category whose role is of prime importance for *M. tuberculosis* physiology and virulence^5–10^. In a previous work we have shown that σ^E^-mediated transcription is inhibited upon interaction with Rv1222, a protein encoded by the gene immediately downstream of *sigE*^11^. Amino acid sequence analysis of Rv1222 revealed a HXXXCXXC motif that is typical of the zinc-associated anti-sigma factor (ZAS) family^12,13^. Additionally, σ^E^ and Rv1222 interaction was shown to be redox dependent, and σ^E^ could also be released from Rv1222 as a result of a PknB-dependent phosphorylation of Rv1222 under surface stress conditions^14^. Taken together, all these observations led to the conclusion that Rv1222 is a **r**egulator of **s**igma **E** factor and therefore was named RseA.

However, in a recent paper Rudra and colleagues have called into question the role of Rv1222 as an anti-σ^E^ factor^15^. They found that the interaction between σ^E^ and Rv1222 does not inhibit open-complex formation mediated by RNAP. Rv1222 was shown to interact with *M. tuberculosis* RNAP core enzyme but not DNA polymerase and bind non-specifically to DNA through its C-terminal region. In *in vitro* transcription experiments Rv1222 was able to inhibit transcription regardless the source of RNAP, for inhibition occurred by using holo RNAP or core RNAP coming either from *M. tuberculosis*, *Escherichia coli* or *Bacillus subtilis*. Additionally, they observed a growth rate reduction in both *Mycobacterium smegmatis* and *E. coli* upon inducible expression of Rv1222 that reversed when the C-terminal region of Rv1222 was removed. Such a growth defect was accompanied with a reduction of the amount of total RNA. Based on these results, they proposed a novel mechanism of transcriptional regulation according to which Rv1222 inhibits transcription by anchoring RNA polymerase onto DNA and slowing down its movement along DNA during elongation.

In this paper, we have performed a series of experiments using a genetic approach to better characterize the role of Rv1222 in *M. tuberculosis* physiology.

## Experimental Procedures

### Bacterial strains, media and growth conditions

*E. coli* strain DH5α was grown in Luria broth (Difco) at 37 °C. When required, antibiotics were added at the following concentrations: kanamycin 50 µg/ml, hygromycin 150 μg/ml.

*M. tuberculosis* H37Rv was grown in either Middlebrook 7H9 liquid medium or Middlebrook 7H10 solid medium (Difco) supplemented with 0.05% Tween 80 and ADN (2% glucose, 5% bovine serum albumin, 0.85% NaCl). *M. tuberculosis* liquid cultures were grown in roller bottles at 37 °C. Plates were incubated at 37 °C in sealed plastic bags. When required, antibiotics were added at the following concentrations: kanamycin 20 µg/ml; streptomycin 20 μg/ml; and hygromycin 50 µg/ml.

All the strains tested in this work are listed in Table 1.

**Table 1:** List of *M. tuberculosis* strains used in this work.

*M. tuberculosis* strains	Relevant genotype or description	Reference
H37Rv	parental strain	PHRI collection
TB340	H37Rv *ΔsigE_Δrv1222*	This work
ST28	H37Rv *ΔsigE* Hyg^R^	^9^
ST29	ST28 *ΔsigE::sigE::rv1222* Hyg^R^ Km^R^	^9^
TB514	TB340 *ΔsigE_Δrv1222*::*sigE* Km^R^	This work
TB508	TB340 *ΔsigE_Δrv1222*::*sigE*::*rv1222* Km^R^	This work
TB515	H37Rv with pMV261::P_hsp60_*rv1222* Km^R^	This work
TB516	TB340 *ΔsigE_Δrv1222* with pMV261::P_hsp60_*rv1222* Km^R^	This work

*M. tuberculosis* was handled and cultivated in a biosafety level 3 (BL3) laboratory.

### DNA manipulation

All recombinant DNA techniques were performed according to standard procedures using *E. coli* DH5α as the initial host. DNA restriction and modifying enzymes were obtained from New England Biolabs and used according to the manufacturer’s recommendations. Primers used are shown in Table S1.

### RNA extraction and quantitative RT-PCR

RNA extraction and quantitative reverse transcription real-time PCR (RT-PCR) were performed using Sybr Green Master Mix (Applied Biosystems) as previously described^16^. Results were normalized to the amount of *sigA* mRNA^17^. RNA samples that had not been reverse transcribed were included in all experiments to exclude significant DNA contamination. For each sample, melting curves were used to confirm the purity of the amplification products. Experiments were performed at least twice, starting from independent biological samples. Primers used for quantitative real-time PCR are described in Table 2.

**Table 2:** Oligonucleotides used for quantitative RT-PCR assays.

Gene	Forward primer	Reverse primer	bp
*sigA*	5′-CCATCCCGAAAAGGAAGACC-3′	3′-AGGTCTGGTTCAGCGTCGAG-5′	209
*sigB*	5′-GTCTATCTGAACGGCATCGG-3′	3′-CCGCCTCGCCATCACGCAC-5′	216
*sigE*	5′-CGAAGGCTGGCTACACCGCA-3′	3′-GCAGGTCAGGTCCCAGCC-5′	199
*sigH*	5′-CCGGATACTGACCAACACCT-3′	3′-CGCTTCTAACGCTTCGACTT-5′	157
*sigL*	5′-GTGGCTCGTGTGTCGGGCG-3′	3′-CGAACCGACCACATTGCG-5′	276
*mprA*	5′-AACGCGCTGGAAGTCTACGT-3′	3′-GGCGAACGACATCAGCACAA-5′	242
*pimA*	5′-GTTGTTCCTGGGTCGCTACG-3′	3′-CACAATGCCGAAACTCTCACC-5′	265
*pspA*	5′-GCATCGTTGCGGTCGATGAG-3′	3′-GCGGATCTGTTCCAACCGTG-5′	190
*rseA*	5′-CAGTTCCGTTCCACCGAGCA-3′	3′-GGTGGACAACGCGGGATCT-5′	230

### Construction of a *ΔsigE_Δrv1222* null mutant in *Mycobacterium tuberculosis*

**Figure 1:**
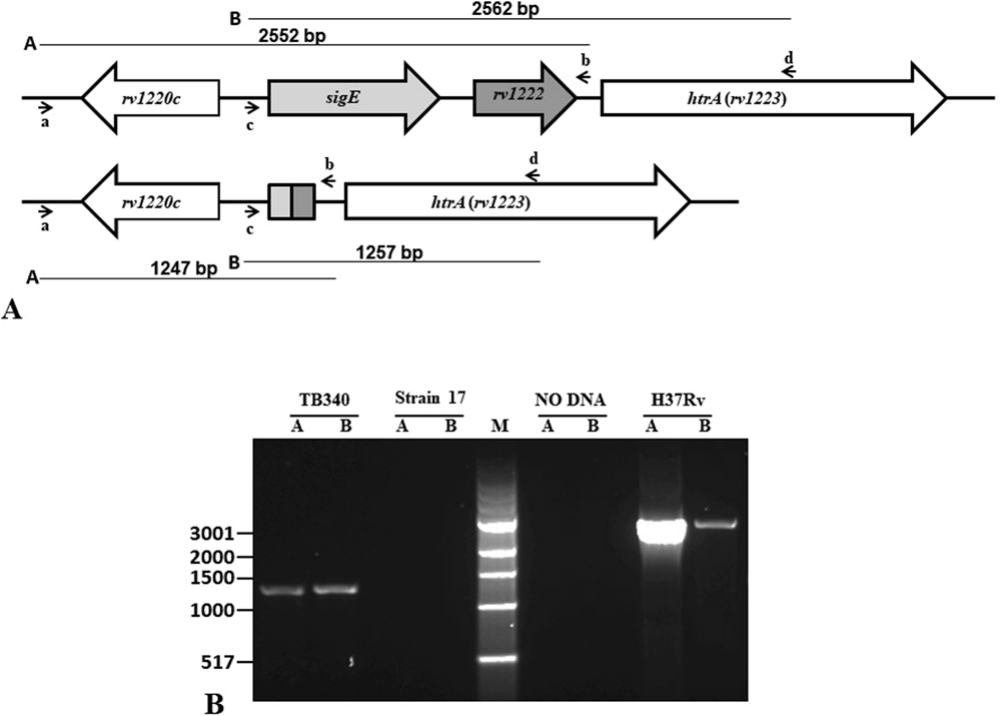
Construction of the *ΔsigE_Δrv1222* mutant *in M. tuberculosis*. (**A**) Schematic representation of the *sigE-rv1222* chromosomal location, and its structure after the correct deletion. Arrows represent primers used for PCR to confirm the deletion. The black lines above and below the chromosomal maps represent the amplification products predicted for primers a/b ((RP1520/RP1521, couple A) or primers c/d (RP1522/RP1523, couple B) in the wt and mutant strain. (**B**) Agarose gel of PCR products obtained amplifying the genomic DNA of H37Rv and two putative mutants with primer couples A and B. One of the recombinant strain that gave products of the expected size was named TB340. M: 1Kb ladder (NEB). Uncropped image of the gel is presented in Supplementary Fig. S1.

An unmarked *ΔsigE_Δrv1222* deletion mutant was constructed in *M. tuberculosis* according to a published method^18^. Two DNA regions, one upstream *sigE* and one downstream *rv1222* were amplified by PCR and sequentially cloned into p1NIL as ScaI/HindIII and HindIII/PacI fragments respectively (Fig. 1A). The *rv1221* upstream region (1012 bp) was amplified by RP1413 and RP1414 while the downstream region (1037 bp) was amplified by RP1415 and RP1416. A *lacZ-sacB-hyg* cassette from pGOAL19 was then introduced as a PacI fragment in the resulting vector to obtain the final suicide plasmid pSA23.1 that was electroporated into *M. tuberculosis*^19^. Transformants were selected on plates containing both kanamycin and hygromycin. The occurrence of single crossover was confirmed by PCR (data not shown). One mutant with the correct integration of pSA23.1 was grown in the absence of any drug to allow a second homologous recombination^20^. Recombinants were isolated as white colonies on plates containing sucrose and X-gal and the occurrence of a double crossover leading to *sigE* and *rv1222* deletion was confirmed by PCR screening with the couple of primers RP1520/RP1521 (couple A) and RP1522/RP1523 (couple B) (Fig. 1B). These primer couples amplify two DNA regions whose length is 2552 bp and 2562 bp respectively in the wt strain, and 1247 bp and 1257 bp respectively in the correct mutant. One strain with the proper chromosomal structure was named TB340.

### Complementation of *M*. *tuberculosis* TB340

To complement TB340 with *sigE*, a 1309 bp fragment containing the whole *sigE* gene and 535 bp upstream with the *sigE* promoter^11^ was amplified by RP1917 and RP1603, and then cloned into the integrative vector able to stably integrate into the single phage *attB* site in the *M. tuberculosis* genome^21^. The resulting plasmid, named pFRA250, was then electroporated into TB340 and transformants were selected on Km plates. The resulting strain was named TB514. To complement TB340 with both *sigE* and *rv1222*, a 1488 bp fragment containing the chromosomal region that includes the *sigE* promoter, *sigE* and *rv1222* was amplified by RP1917 and RP1918 and then cloned into pMV306. The resulting plasmid was named pFRA242 and was introduced into TB340 by electroporation. Transformants were selected on Km plates. The resulting strain was named TB508.

### Construction of *M*. *tuberculosis* strains over-expressing *rv1222*

*rv1222* was amplified by primers RP1942bis and RP1943 and then cloned into the replicative plasmid pMV261 under the P_*hsp60*_ transcriptional control. The resulting plasmid, named pLCM1 was electroporated into H37Rv and TB340 to obtain TB515 and TB516 respectively.

## Results

### Effect of Rv1222 on *M. tuberculosis* growth rate

**Figure 2:**
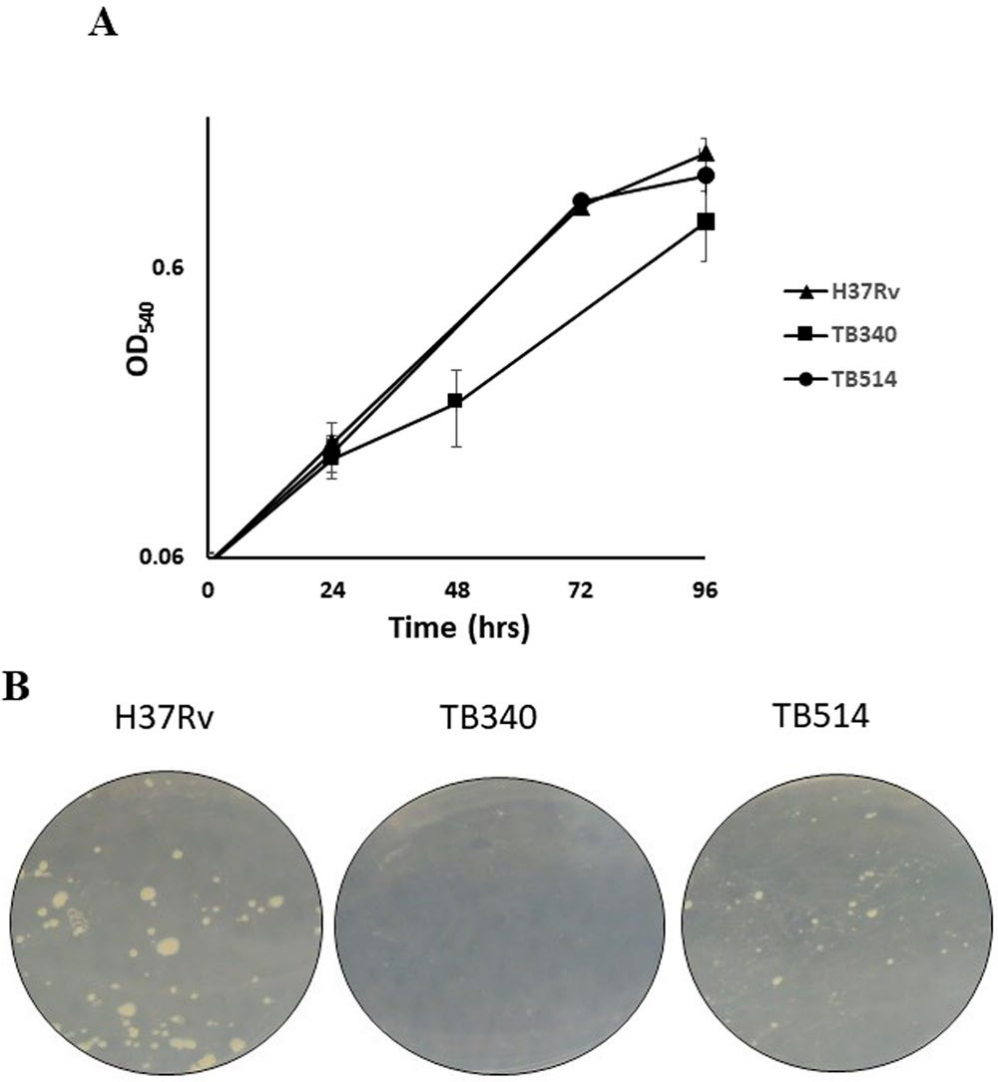
Growth of *M. tuberculosis* mutant strains in liquid and solid medium. (**A**) Growth curves in liquid medium. Bacteria were grown in 7H9 ADC at 37 °C in rolling bottles; OD_540_ was recorded at regular intervals up to 96 hrs; results represent the average of three independent experiments; (**B**) Growth in solid medium. Bacteria were inoculated in Middlebrook 7H10 plates and incubated at 37 °C for 21 days.

**Figure 3:**
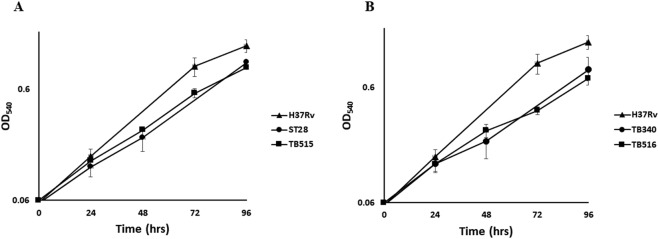
Growth curve profiles of *M. tuberculosis* overexpressing Rv1222. Bacteria were grown in 7H9 ADC at 37 °C in rolling bottles; OD_540_ was recorded at regular intervals up to 96 hrs. Results represent the average of three independent experiments.

Rudra and coll^15^ proposed that overexpression of Rv1222 leads to a general decrease of RNA transcription and a consequent reduced growth rate, therefore hypothesizing that deletion of this gene (constitutively expressed in physiological conditions) would result in an increase of *M. tuberculosis* growth rate. To challenge this hypothesis we compared the growth of H37Rv, TB340 (missing both *rv1222* and *sigE*) and TB514 (TB340 complemented with a single copy of *sigE*, thus missing only *rv1222*). As clearly shown in Fig. 2A, H37Rv and TB514 (devoid of *rv1222*) grew with the same rate, whereas the double mutant grew slightly slower confirming the importance of σ^E^ on the growth of *M. tuberculosis*^9^. The same data could be observed when bacteria were grown on solid media (Fig. 2B). The deletion of *rv1222* thus did not lead to an increase of *M. tuberculosis* growth rate. Subsequently, we analyzed whether *rv1222* overexpression would decrease *M. tuberculosis* growth rate, as already observed for *M. smegmatis* and *E. coli* by Rudra and coll.^15^. For that purpose, we compared the growth profile of H37Rv, ST28 (single *sigE* mutant) and TB515 (an H37Rv derivative where *rv1222* expression was sevenfold higher than in wild type, see below). As shown in Fig. 3A, overexpression of *rv1222* indeed slightly decreased *M. tuberculosis* growth rate whose profile was comparable to that of ST28. This could be explained by two different reasons: either (i) a decreased RNA transcription rate which would lead to a decreased growth rate, as stated by Rudra and coll.^15^, or (ii) a reduced activity of σ^E^ due to the inhibitory action on σ^E^ carried out by an increased amount of Rv1222 that consequently would implicate a growth rate reduction as observed for the *sigE* mutant. To discriminate between these two hypotheses, we overexpressed Rv1222 in TB340, which is devoid of both *sigE* and *rv1222* (TB516). If overexpression of Rv1222 directs a reduction of the bacterial growth rate, we would expect an additional slowdown in the growth of TB516. However, as shown in Fig. 3B, Rv1222 overexpression in this strain had no effect on its growth, suggesting that the sole consequence of overexpressing Rv1222 is to inhibit σ^E^ activity, leading consequently to a slightly reduced growth rate.

### Deletion of *rv1222* causes specific induction of genes whose expression is controlled by σ^E^

**Figure 4:**
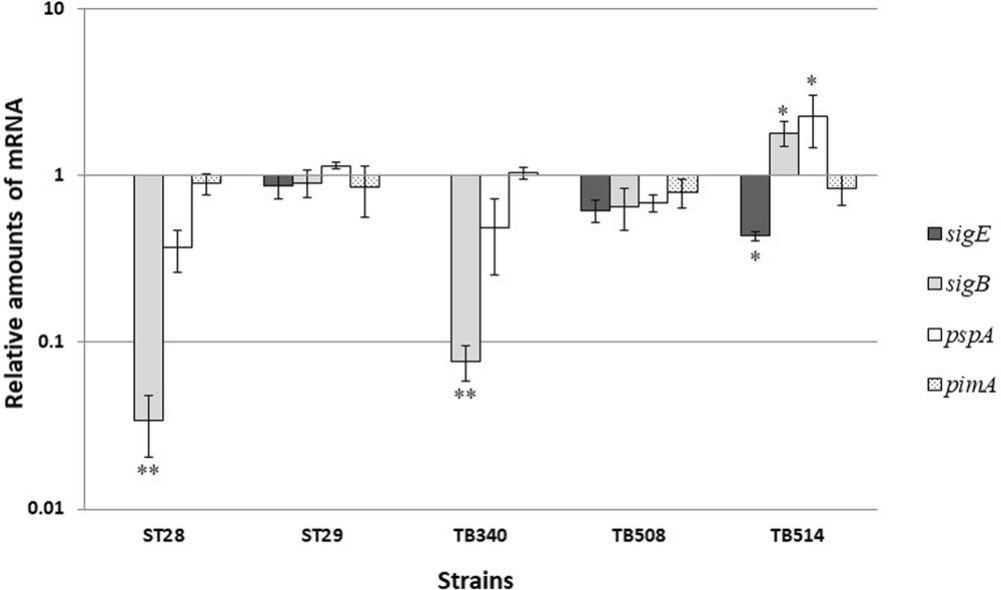
Relative amounts of mRNA level in different H37Rv mutants. The values are expressed as the ratio between the number of cDNA copies detected by quantitative RT–PCR in samples obtained from exponentially growing cultures of the different mutants and their parental strain H37Rv. Data were normalized to the level of *sigA* cDNA that represented the internal invariant control. The reported values derive from two independent experiments. *P < 0.05 **P < 0.01 versus wt (Student’s *t*-test).

If Rv1222 acted as a σ^E^-specific anti-sigma factor, we would expect to detect an upregulation of those genes whose expression is regulated by σ^E^ when Rv1222 is absent. To investigate this hypothesis, we focused our attention on *sigB* and *pspA*. While *sigB* basal level of expression is almost totally σ^E^ dependent, *pspA* expression is known to be σ^E^ dependent only under stress conditions activating σ^E^ ^5,9^. Indeed, in a genetic background missing σ^E^ (TB340 and ST28) the expression of *sigB* and *pspA* was repressed compared to the wild type, of about 10 times and 2 times respectively (Fig. 4). As expected, when the single mutant ST28 was complemented by reintroducing a copy of *sigE* in its genome, *sigB* and *pspA* expression was totally restored. When the double mutant TB340 was complemented by reintroducing a single copy of both *sigE* and *rv1222* on the genome, we obtained a partial but nevertheless clear and significant complementation of the phenotype. However, when this mutant was complemented with the sole *sigE*, both *sigB* and *pspA* were expressed at a higher level than the wild-type, suggesting that in the absence of Rv1222 σ^E^ was more active, despite of a 50% reduction in the amount of its transcript levels (due to partial complementation, Fig. 4). To verify whether these results were not due to an artifact, we also measured the expression of *pimA* (*rv2610c*), an essential gene required for the biosynthesis of phosphatidyl-myoinositol mannosides^22^ whose regulation is not controlled by σ^E^. As shown in Fig. 4, *pimA* expression level did not undergo any significant variation in all of the genotypic backgrounds tested. Taken together these data indicate that the absence of Rv1222 specifically increases the activity of σ^E^.

### Overexpression of Rv1222 specifically decreases expression of σ^E^ -dependent genes

**Figure 5:**
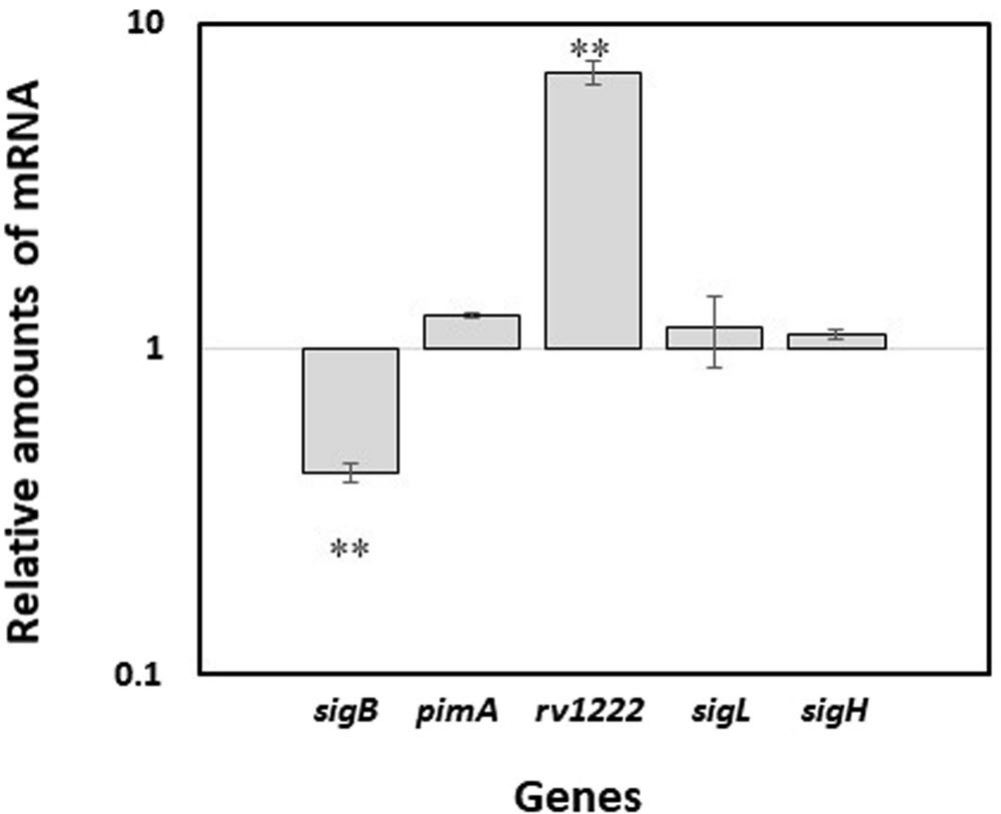
Effects of *rv1222* over-expression on relative amount of mRNA levels of different genes. Values are expressed as a ratio between the number of cDNA copies detected by quantitative RT–PCR in samples obtained from exponentially growing TB515 (overexpressing *rv1222*) and its parental strain H37Rv. Data were normalized to the level of *sigA* cDNA, that represented the internal invariant control. The reported values derive from two independent experiments. **P < 0.01 versus wt (Student’s *t*-test).

To further confirm that Rv1222 specifically modulates σ^E^ activity, we compared the expression levels of a panel of genes in H37Rv and TB515 (H37Rv overexpressing *rv1222*). As shown in Fig. 5, *rv1222* was induced sevenfold in TB515 compared to the wild type. The expression of *sigH*, *sigL*, and *pimA*, whose transcription does not depend on σ^E^ ^23,24^, was not affected by *rv1222* overexpression, whereas we could observe a reduction of *sigB* mRNA level, consistent with the role of Rv1222 as an anti-σ^E^ factor, as the upregulation of *rv1222* would reduce the activity of σ^E^ that is responsible for the basal transcriptional level of *sigB*.

## Discussion

Rudra and coll.^15^ recently challenged the hypothesis that Rv1222 represents a specific anti-σ^E^ factor and proposed a different mechanism of action through a series of *in vitro* experiments, according to which Rv1222 binds to both RNAP and DNA and reduces transcription rate. To corroborate this hypothesis the authors showed that overexpression of this protein in *E. coli* and *M. smegmatis* led to a reduction of total mRNA production and consequently of their growth rate, hence the proposal that the deletion of *rv1222* might increase mycobacterial growth rate. In this manuscript, we described a series of genetic and physiologic experiments challenging this hypothesis.

### Neither overexpression nor deletion of *rv1222* have any role in modulating *M. tuberculosis* growth rate

Using different *M. tuberculosis* mutants, we first showed that an *M. tuberculosis* strain devoid of *rv1222* does not exhibit an increase in growth rate. Then we showed that a sevenfold increase in *rv1222* expression slowed down *M. tuberculosis* growth only in a genetic background where active σ^E^ was present. In fact, such a growth profile was similar to that observed for a *sigE* mutant, suggesting that such a decrease relied on an inhibitory action on σ^E^ activity caused by an increased amount of anti-sigma rather than on a direct, σ^E^-independent, activity of Rv1222 on RNAP. This was also confirmed by RT-PCR experiments showing that Rv1222 overexpression led to a repression of *sigB* (whose expression is totally controlled by σ^E^)^9^, but not of *pimA*, *sigL* or *sigH*, whose expression does not depend on σ^E^ ^22–24^. We were not able to explain the apparent discrepancy of our data with those shown by Rudra and coll., however in their experiments they used expression systems based on acetamide- or IPTG- inducible promoters (in *M. smegmatis* and *E. coli* respectively). These systems are known to produce large amounts of proteins^25,26^. To perform our experiments, we preferred to use a promoter that expresses the gene at “more physiological” levels (at the best of our knowledge no data reporting *rv1222* inductions higher than 2.2 folds have been published). Perhaps the amount of Rv1222 obtained upon induction by Rudra and coll.^15^ was far above the physiological levels thus resulting in non-specific toxicity of the protein. Unfortunately, they did not report the fold induction obtained in their experiment to address this hypothesis.

### Deletion or overexpression of *rv1222* specifically modulate the expression of genes under σ^E^-transcriptional regulation

As already mentioned, Rudra and coll.^15^ hypothesized that Rv1222 down-modulate transcription in a non-specific manner. If this were true, its overexpression or deletion of its structural gene should affect most of the *M. tuberculosis* gene transcription levels.

However, after overexpressing Rv1222 we could observe transcriptional repression of the σ^E^-dependent gene *sigB*, but not of the σ^E^-independent genes *sigL*, *sigH*, and *pimA*. Additionally, partial complementation of the repression of the σ^E^-dependent genes *pspA* and *sigB* was obtained in the double *sigE-rv1222* mutant upon reintroduction of both *sigE* and *rv1222*, whereas the reintroduction of the sole *sigE* in the double mutant led to increased expression of *pspA* and *sigB*, despite a reduced amount of *sigE* mRNA detected in this genetic background as compared to the wild-type. On the other hand, the mRNA level of the *sigE* independent gene *pimA* remained the same in all the tested strains. These observations suggested that Rv1222 is required to modulate σ^E^ activity specifically. The reduction of *sigE* expression in a genetic background devoid of *rv1222* could be explained by the presence of some still unknown feedback control mechanisms able to down-modulate its expression to compensate the increased activity of σ^E^ caused by the loss of post-translational control.

Taken together these data strongly support the role of Rv1222 as an anti-sigma factor regulating σ^E^ and are in contrast with those published by Rudra and coll. These authors showed that Rv1222 couldn’t inhibit open complex formation in an EMSA assay. We noted however that a control without σ^E^ was lacking in their experiments in order to ensure that the shift observed was not due to non-specific binding of RNA polymerase to the probed DNA fragment. In addition, we also noted that these authors used high concentrations of RNA polymerase in their *in vitro* experiments, a condition that may well induce non-specific interactions of this protein with DNA. There is no doubt however, that Rv1222 inhibits *in vitro* transcription, regardless of what sigma factor is used, because it binds to both RNA polymerase and DNA through its C-terminal region, however our data strongly suggest that the situation is completely different *in vivo*, where Rv1222 is able to inhibit only σ^E^-mediated transcription. In conclusion, our *in vivo* genetic analyses support a direct role for Rv1222 (RseA) as a specific anti-SigE factor and are in line with our previously published *in vitro* experiments^11,14^.

## Supplementary information

Table S1 and Figure S1
