# Publisher Correction: Assessing the role of Rv1222 (RseA) as an anti-sigma factor of the *Mycobacterium tuberculosis* extracytoplasmic sigma factor SigE

**DOI:** 10.1038/s41598-019-54232-9

**Published:** 2019-11-22

**Authors:** Francesca Boldrin, Laura Cioetto Mazzabò, Saber Anoosheh, Giorgio Palù, Luc Gaudreau, Riccardo Manganelli, Roberta Provvedi

**Affiliations:** 1 Department of Molecular Medicine, University of Padova, Padova, Italy; 2 Département de biologie, Université de Sherbrooke, Sherbrooke, QC J1K 2R1 Canada; 3 Department of Biology, University of Padova, Padova, Italy; 4 Present Address: Institute of Infectious Disease and Molecular Medicine UCT, Cape Town, South Africa

Correction to: *Scientific Reports* 10.1038/s41598-019-41183-4, published online 14 March 2019

In the original version of this Article, the author Laura Cioetto Mazzabò was incorrectly indexed. This error has now been corrected.

