# Supplementary material for: Assessing the role of Rv1222 (RseA) as an anti-sigma factor of the Mycobacterium tuberculosis extracytoplasmic sigma factor SigE
Source: Sci Rep. 2019 Mar 14;9:4513. doi: 10.1038/s41598-019-41183-4 (PMC6418294; doi:10.1038/s41598-019-41183-4)
Supplement: Supplementary file 1 — Table S1 and Figure S1 [file 41598_2019_41183_MOESM1_ESM.pdf]

Assessing the role of Rv1222 (RseA) as an anti-sigma factor of the *Mycobacterium tuberculosis* extracytoplasmic sigma factor SigE

Francesca Boldrin[1], Laura Cioetto Mazzabò[1], Saber Anoosheh[1§], Giorgio Palù[1], Luc Gaudreau[2], Riccardo Manganelli[1], Roberta Provvedi[3]*

[1]Department of Molecular Medicine, University of Padova, Padova, Italy; [2]Département de biologie, Université de Sherbrooke, Sherbrooke, QC, J1K 2R1, Canada; [3]Department of Biology, University of Padova, Padova, Italy.

[§]**Present address:** Institute of Infectious Disease and Molecular Medicine
UCT

Table S1. Primers used in the study

PRIMER	SEQUENCE 5'-3'	PURPOSE
RP1413	AATAGTACTAGCAGACGCAAAATCGCC	*sigE* upstream region amplification, upper primer
RP1414	AATAAGCTTGATTCCGTATTCCCAACC	*sigE* upstream region amplification, lower primer
RP1415	AATAAGCTTCTGACGGCGATGGCGGGA	*rv1222* downstream region amplification, lower primer
RP1416	TTGTTAATTAACAAGTCGGTCTTGGGGTC	*rv1222* downstream region amplification, upper primer
RP1917	AAGGCCTGATATCGATGGTGGTCAAGACG	TB340 complementation with *sigE* + upstream region, upper primer
RP1603	CAGCTGTCAGCGAACTGGGTTGACGTG	TB340 complementation with *sigE* + upstream region, lower primer
RP1918	AAGGCCTTTGTCCGTGTCCACCCTAGTATC	TB340 complementation with *sigE_rv1222* + upstream region, lower primer
RP1942bis	AAGGCCTCTGCCGACCCCGGAAGCGTG3	*rv1222* amplifcation for its over-expression with plasmid pLCM1, upper primer
RP1943	GGAATTCACAAGTGGCTCACCGGCTA	*rv1222* amplifcation for its over-expression with plasmid pLCM1, lower primer
RP1520	GTTCGATGGCCGCCTCTCTG	PCR screening *ΔsigE_Δrv1222* mutant
RP1521	CCATGACTCGCAGCAACCCC	PCR screening *ΔsigE_Δrv1222* mutant
RP1522	ACTTTGACTCGATATTCCCTG	PCR screening *ΔsigE_Δrv1222* mutant
RP1523	GACCCGTACCTTGCTGGA	PCR screening *ΔsigE_Δrv1222* mutant

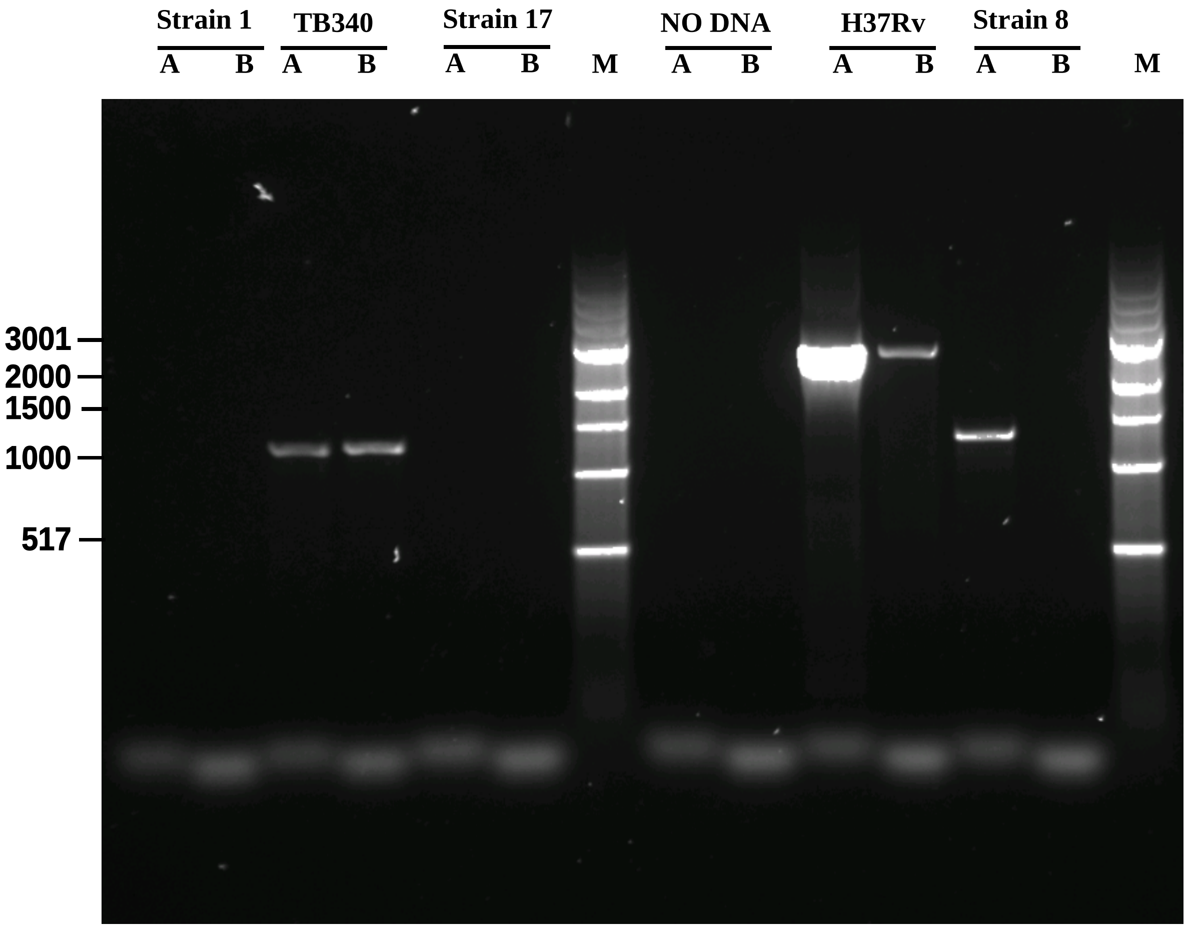

Strain 1
TB340
Strain 17
NO DNA
H37Rv
Strain 8
A B A B A B M A B A B A B M
3001
2000
1500
1000
517

Figure S1
